# Adjustable Compliance Soft Sensor via an Elastically Inflatable Fluidic Dome

**DOI:** 10.3390/s21061970

**Published:** 2021-03-11

**Authors:** Xingtian Zhang, Jun Kow, Dominic Jones, Greg de Boer, Ali Ghanbari, Ahmad Serjouei, Pete Culmer, Ali Alazmani

**Affiliations:** 1 School of Mechanical Engineering, University of Leeds, Leeds LS2 9JT, UK; mnxzha@leeds.ac.uk (X.Z.); J.Kow@leeds.ac.uk (J.K.); D.P.Jones@leeds.ac.uk (D.J.); G.N.deBoer@leeds.ac.uk (G.d.B.); A.Ghanbari@leeds.ac.uk (A.G.); P.R.Culmer@leeds.ac.uk (P.C.); 2 Department of Engineering, School of Science and Technology, Nottingham Trent University, Nottingham NG11 8NS, UK; ahmad.serjouei@ntu.ac.uk

**Keywords:** soft sensor, tactile sensing, variable stiffness, adjustable compliance, soft robotics

## Abstract

Soft sensors are essential for robotic systems to safely interact with humans and the environment. Although significant research has been carried out in the field of soft tactile sensing, most of these sensors are restricted to a predefined geometry and a fixed measurement range, hence limiting their application. This paper introduces a novel approach to soft sensing by proposing a soft load-sensing unit with an adjustable mechanical compliance achieved using an elastically inflatable fluidic dome. The sensor consists of a three-dimensional Hall-effect sensor, above which is a magnet whose movement is modulated by an intermediate elastomeric dome structure. Sensor configurations were designed and fabricated using three different silicone rubbers to cover ‘00–10’ and ‘20A’ durometer shore hardness scales. We demonstrated that the compliance of the sensor could be dynamically tuned by changing the internal pressure of the inflatable fluidic dome in all configurations. We performed finite element simulations to determine the reaction force of the sensor under load as well as the stresses within the internal structural behavior, which are not possible to capture experimentally. The proposed soft sensor has the potential to be readily adapted for use in various soft robotic applications of differing size, compliance range, and safety requirements.

## 1. Introduction

In recent years, soft robotics have been developed with reduced complexity, greater adaptability, and for a safer interaction with delicate objects, humans, or unstructured environments owing to their intrinsic material compliance [1,2,3]. However, technical challenges remain to be addressed for soft robotics to reach a human-level performance in terms of material level compliance, soft sensing, and soft actuation [4,5]. Soft sensing is essential for creating fully functional and practical soft robots. Several soft tactile sensors have been developed to obtain feedback with a high level of accuracy using high-compliance materials [6,7]. These soft tactile sensors measure force indirectly by transducing optical [8], resistive [9], capacitive [10], inductive [11], and magnetic [12] properties into force. For instance, Ohmura et al. [13] developed a soft optical tactile sensor by detecting the concentration of the scattered light in a soft urethane foam when deformed. This design improved adaptivity of soft sensors on curved surface. In another study, Hammond et al. [14] presented a soft tactile sensor that used conductive fluid-filled micro-channels embedded in soft elastomer layers. This sensor array was miniaturized to micrometer scale, expanding its application to microgrippers in surgery. Later, Sun et al. [15] proposed an ionic skin by creating a soft conductive hydrogel consisting of a dielectric elastomer, which could be used as a large-area stretchable sheet of distributed sensors. This sensor achieved high optical transparency and low stiffness using hydrogel, which improved comfort on human wearable sensors. Kim et al. [16] reported an all-carbon piezo-capacitive tactile sensor by encapsulating carbon nanotube fabrics in PDMS substrate. The sensor was highly sensitive, wearable, highly stretchable, and multi-stimuli-responsive.

Existing soft tactile sensors are limited by their complicated fabrication, fragile materials, structural complexity, and insufficient performance on measurement range and sensitivity. To address above disadvantages, researchers have fabricated magnetic-based tactile sensors by embedding magnet(s) in a deformable matrix [17] featuring durable materials, low-cost and simple fabrication, high accuracy, and bandwidth. When an external force is applied, the matrix and magnet are displaced, causing a change in the magnetic field detected by the sensor. These soft magnetic sensors contribute to a safe interaction between robots and the human since they provide a greater flexibility in their application. For example, magnetic-based soft tactile sensors can be integrated into a robotic hand to help grasp fragile objects with the proper force to manipulate them without causing damage [18,19]. Goka et al. [20] produced a robust, low-cost soft tactile sensor by injecting a magnet inside a soft elastomer, which sat on a substrate layer with four Giant Magneto Resistance (GMR) elements and four chip inductors. The displacement of the magnet was detected by the GMRs, from which a force vector was calculated. For more accurate measurement and further cost reduction, a tactile sensor was developed using a three-dimensional (3D) Hall-effect transducer [21], which was employed to locate the relative movement of the magnet. Considering the displacement and elasticity of the substrate into account, the magnitude of the force was then calculated. Yousseefian et al. [22] applied a bioinspired soft spherical shell onto the conventional soft tactile sensors to mimic the ridges in the skin, and used a Hall-effect sensor and magnet to detect the displacement and force. In our previous work [23], we developed a three-axis soft tactile sensor which consisted of a 3D Hall-effect sensor and a magnet embedded onto a silicone elastomer. This design demonstrated a low-cost, easy-to-manufacture, and high-sensitivity soft sensor for robotic applications.

Controllable compliance is also essential for functional soft robots to facilitate multi-task movement, adaptable locomotion, and dexterous manipulation of various objects. The deformable nature of soft sensors and their fixed stiffness makes it difficult to build a controllable and dynamic measurement system for soft tactile sensors [21,22,23]. This is even more critical for elastic sensors in contact with rigid surfaces where overloading could easily lead to sensor damage. Equally, developing soft sensors with repeatable and changeable levels of stiffness is crucial to the performance (e.g., sensitivity, measurement range) of soft robots under higher levels of forces [23]. In the literature, the relevant technologies for this purpose are mostly based on the use of external pneumatic sources, electromagnetic fields, and temperature to control the compliance of the soft structure, using techniques such as particle jamming [24], electrorheological/magnetorheological fluids [25], and shape memory polymers [26], respectively. Variable stiffness and actively controlled compliance offer potential solutions to the problem of dynamic measurement, an area largely overlooked in the current state-of-the-art. Some researchers in soft robotics have found that the application of variable compliance is also essential in soft actuators [27]. Several methods have been provided to achieve variable and adaptive stiffness [24,28] in soft actuators which can also be modified in similar soft sensor structures. For example, Nagase et al. [29] proposed a variable stiffness robotic hand using pneumatic control. A soft rubber actuator was applied to adjust the stiffness of a robotic hand by changing the air pressure inside a rubber actuator. Another method, used by Shintake et al. [30], was a variable stiffness actuator consisting of a soft dielectric elastomer actuator and a low-melting-point alloy. Existing solutions for variable stiffness are most related to soft actuators but not adaptive for soft sensors, as they require either a complicated actuation system or additional materials and features for sensing. A simple and effective way to achieve variable stiffness soft sensor is needed.

In this paper, we demonstrate a novel variable compliance load-sensing unit in which the sensing and the controllable compliance are provided by a single system, thus the robotic structure is drastically simplified. The sensor can adjust its compliance by changing the pressure inside its elastically inflatable fluidic dome. This can be harnessed to alter the dome’s load-carrying capacity as well as the sensing range. Section 2 illustrates the concept including the structure design and analysis, material selection, finite element analysis (FEA) simulation, and fabrication process. Section 3 then describes the experiments that were conducted to validate and characterize the performance of the proposed sensor. In Section 4, the potentials and limitations of the research are discussed. Finally, Section 5 concludes the research and potential applications for the proposed sensor.

## 2. Materials and Methods

### 2.1. Structure Design and Analysis

**Figure 1:**
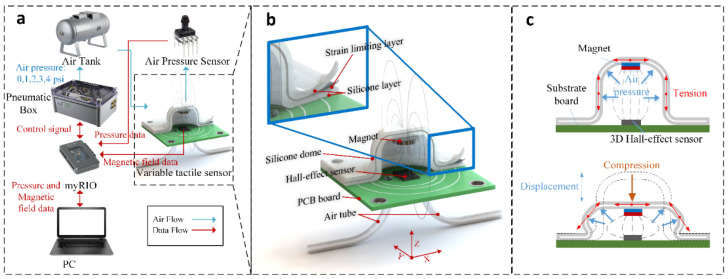
Overview of the soft sensor with an adjustable compliance: (**a**) A closed-loop control system, which is connected to a pneumatic circuit with feedback control of air pressure for altering the air pressure inside an elastically inflatable dome with an embedded magnet mounted on top of a Hall-effect sensor. This system is connected to a real-time control system to record data from the Hall-effect and air pressure sensors. (**b**) The soft sensor consists of elastically inflatable fluidic dome, embedded magnet, and a Hall-effect sensor soldered on a PCB board. (**c**) The elastomeric fluidic dome is reinforced by a sheet of inextensible embroidery fabric to control the expansion of the structure under variable air pressure conditions. With known overall stiffness of the structure, the sensing system can be calibrated to measure input force from the displacement of the magnet detected by the Hall-effect sensor during mechanical compression.

The proposed soft load-sensing unit for tactile sensing, described herein as a soft sensor, consists of three main components: a Hall-effect sensor, a magnetic source, and an anisotropic flexible dome-structure that is elastically inflated by pressurized air, as shown in Figure 1. The dimension of the sensor is 30 mm × 30 mm × 12 mm, and the diameter of the dome is 16 mm. The dome has a void cavity of 12 mm diameter with 2 mm wall of silicone rubbers. The design of the inflatable dome can be tailored, together with the mechanical properties of the elastomeric material, to realize different compliance levels when the dome is in contact with external objects. Varying levels of compliance were achieved for the dome structure through the use of different silicone elastomers with a range of durometer shore hardness (00–10 to 20 A) and mechanical stiffness (elastic moduli of the order of 10^5^–10^7^ Pa). Moreover, by controlling the internal fluidic pressure level within the dome, this study aims to develop a soft load-sensing unit for tactile sensing in which the compound stiffness of the dome, and therefore its measurement range, can be adjusted in real-time.

For this purpose, a hollow silicone dome was created by fabricating a compliant wall consisting of a strain-limiting layer sandwiched between two elastomeric layers. A permanent magnet is embedded into the upper part of the dome. This inflatable dome was then fixed above a 3D Hall-effect sensor, as illustrated in Figure 1a,b. Figure 1 also shows the fluidic pressure control system used to vary the compliance of the dome. Two air tubes are fed into the void, one to allow the introduction of pressure, the other to facilitate measurement of the internal pressure through use of a pressure transducer. On the application of the air pressure inside the inflatable dome, the strain-limiting layer prevents the dome from excessive inflation, while the structural compliance of the dome increases, as shown in Figure 1c. Through closed-loop control of the internal pressure, the compliance of the dome can be tuned to alter its reaction to the external force and, therefore, the overall measurement range of the soft sensor can be adjusted.

When an external normal force is applied to the dome, the magnet is displaced, causing a variation in the magnetic field detected by the 3D Hall-effect sensor. The change in the magnetic field is related to the displacement of the magnet by:

(1) $$d=\frac{B}{S_{h}},$$

where *S_h_* is the sensitivity of the 3D Hall-effect sensor, *B* is the magnetic field, and d is the displacement of the magnet. The applied external force, F, is then described by:

(2) F=d·K,

where K is the stiffness of the inflatable fluidic dome (which changes dependent on the fluidic pressure within the dome).

### 2.2. Material Selection and Fabrication

The mechanical behavior of the proposed soft sensor is linked to both the selected elastomeric material and the manufacturing process used to fabricate the inflatable dome, since they affect the produced compliance levels required for the sensing application. In addition, compliance and stiffness determine the robustness of the structure to leaks in pressure or structural failure. The stiffness of the chosen material also affects the adjustable measurement range as stiffer material create a larger range with a higher measuring start.

We used silicone rubber at three durometer shore hardness values (Ecoflex 0010, Ecoflex 0050, and Dragon Skin 20, Smooth-On, Inc., Macungie, PA, USA) to produce different mechanical properties for the elastomeric layers. An isotropic and inextensible non-woven embroidery fabric (Cut-away Stabilizer, Sulky, Hawkinsville, GA, USA) was selected as a strain-limiting layer sandwiched between two layers of silicone rubber to constrain the circumferential strain. This fabric is highly permeable, allowing integration with the pre-polymer liquid silicone rubber, thus helping ensure mechanical integrity of the sandwiched layers.

**Figure 2:**
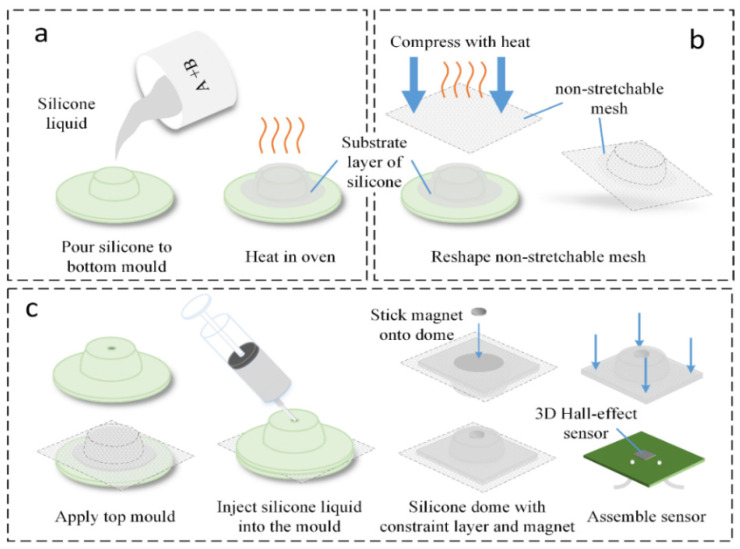
Fabrication of the soft sensor: (**a**) the bottom elastomeric layer is formed by pouring pre-polymer silicone rubber onto the bottom mold and then curing by heating in oven at 45 ℃ for 15 min. (**b**) Thermal pre-shaping of the inextensible strain-limiting fabric layer. (**c**) Fabrication of the top layer by mold-casting and assembly of the dome onto the three-dimensional (3D) Hall-effect sensor PCB.

The fabrication process for the inflatable dome consists of three main steps, as shown in Figure 2. A two-part mold was designed to produce the elastomeric wall of the fluidic dome. In step 1, shown in Figure 2a, silicone rubber precursors were mixed in a 1:1 weight ratio at 2100 rpm for 60 s and degassed at 2100 rpm for an additional 1.5 min (ARE-250, Thinky, Tokyo, Japan), then poured onto the bottom mold and cured at room temperature for 5 h. This stage of the curing process was accelerated using an oven at 45 ℃ for 15 min. Next, as shown in Figure 2b, the two-dimensional strain-limiting fabric was heated using a heat gun until slight heat shrinkage was observed. The fabric was quickly placed in between the top and bottom molds and compressed into the three-dimensional shape of the fluidic dome. This step was performed to achieve an optimum fit and attachment between the fabric and the elastomeric layers in the following step. In step 3, as shown in Figure 2c, silicone rubber precursors were mixed and degassed as in step 1, and then injected into the cavity between the molds. The dome was then cured for 5 h at room temperature (the oven was not used for this stage to allow air to escape from the silicone and reduce defects in the dome). After curing was complete, the dome was removed from the molds and a permanent magnet was glued with Sil-Poxy (Smooth-On, Inc.) onto the internal surface of the dome. The resulting elastomeric dome was affixed onto a PCB with a 3D Hall-effect sensor (MLX90393, Melexis, Ieper, Belgium) to form a fluidic void.

**Figure 3:**
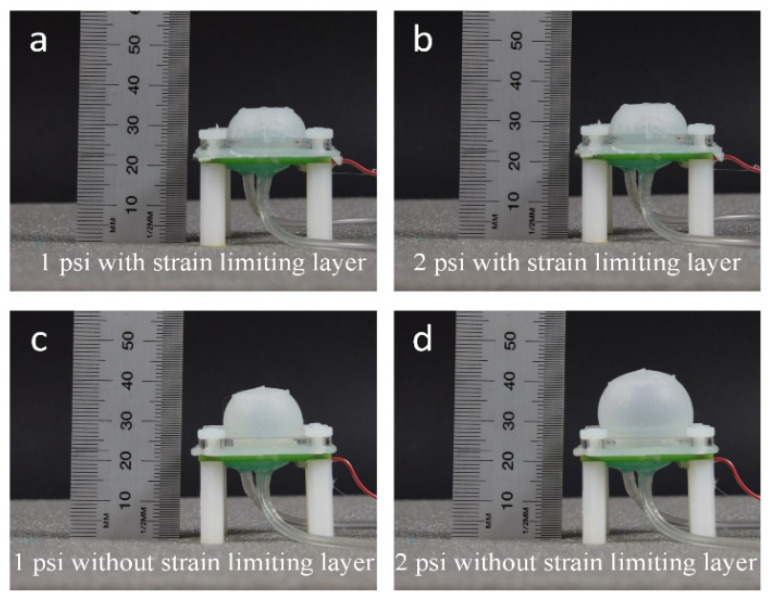
Comparison between structural expansions of the inflatable domes designed for the proposed sensor. The dome with a strain-limiting layer exhibited no deformation when it was pressurized with air to (**a**) 1 psi and (**b**) 2 psi. The prototyped domes without strain-limiting layer showed obvious deformation when pressurized with air to (**c**) 1 psi and (**d**) 2 psi.

The addition of the strain-limiting layer aims to minimize deformation of the fluidic dome under different internal pressures such that deformation of the dome, and resultant movement of the magnet, is dominated by the action of external loads. This facilitates characterization and calibration and reduces the risk of mechanical failure from over-strain of the elastomeric dome. A comparison is shown in Figure 3 between the sensor’s dome with and without a strain-limiting layer. Both samples were fabricated with Ecoflex 0050 as the elastomeric layer and using the same mold. In comparison, the dome with strain-limiting layers has negligible deformation when both samples were under the same internal pressure, exhibiting good characteristics for use as a sensor.

### 2.3. Finite Element Methods

A Finite Element (FE) model of the sensor’s inflatable dome was developed using commercial FE software (ABAQUS standard solver, Simulia, Providence, RI, USA) to characterize the representative reaction force on the sensor during indentation as well as observing the deformation mechanisms within the structure, which is not possible to capture in an experimental setting. The strain and stress distribution contours within the sensor obtained from the simulations can then be used as a design tool for modification of the sensor to suit different applications.

The simulation is performed in two steps: (1) a pressurizing step in which a uniform pressure as boundary condition is applied into the interior of the inflatable dome and (2) the indentation step, in which the pressurized dome was indented by the vertical displacement of a 22 mm diameter rigid indenter onto the top exterior surface of the structure (the initial pressure is maintained within the dome).

**Figure A1:**
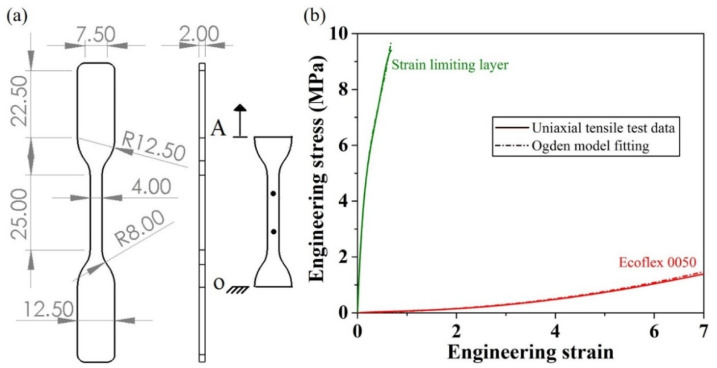
Uniaxial tensile tests: (**a**) Schematics of a sample used for tensile test. The values are in mm. The inset shows the gauge points used to measure the axial strain by using a digital-image-correlation (DIC) method. The horizontal lines at “O” and “A” in the shoulder region indicate where the specimens were gripped at the shoulders of the dog-bone specimens. (**b**) Uniaxial tensile engineering stress-strain graph of Ecoflex 0050 and strain-limiting layer as well as the Ogden model fitted to their respective tensile test data.

The dome sensor and indenter were modeled as axisymmetric deformable geometries and meshed using four-node bilinear axisymmetric quadrilateral elements with hybrid formulation (CAX4RH) within ABAQUS software. A linear elastic model with 1.6 GPa elastic modulus was used in simulations for the indenter, which was 3D-printed using Formlab Clear Standard material [31]. It should be noted that indenter material with such a high stiffness can be considered an un-deformable solid compared to the soft dome sensor. The Ogden constitutive model was employed to represent the behavior of the three materials, namely Ecoflex 0050, Dragon skin 20, and constrain layer. The Ogden model [32] is based on the three principal stretches (*λ*_1_, *λ*_2_, *λ*_3_) and 2·*n* material constants, where *n* is the number of polynomials that constitute the strain energy density function defined as:

(3) $$U=\sum_{i=1}^{n}\frac{2\mu_{i}}{\alpha_{i}{}^{2}}\left({\bar{\lambda }}_{1}{}^{\alpha_{i}}+{\bar{\lambda }}_{2}{}^{\alpha_{i}}+{\bar{\lambda }}_{3}{}^{\alpha_{i}}-3\right)+\sum_{i=1}^{n}\frac{1}{D_{i}}{\left(J-1\right)}^{2i},$$

where U is the strain energy density, and $\mu_{i}$, $\alpha_{i}$ are material constants and $D_{i}$ are incompressible parameters used to indicate volume change. ${\bar{\lambda }}_{i}$ are the deviatoric principal stretches defined as ${\bar{\lambda }}_{i}=J^{\frac{-1}{3}}\lambda_{i}$, where $J=\sqrt{\det\left(B\right)}$ and B is the left Cauchy-Green strain tensor. B is defined as $B={FF}^{T}$ and F is the strain gradient tensor. The initial shear modulus and bulk modulus for the Ogden form are given by $\mu_{0}=\sum_{i=1}^{n}\mu_{i}$, $K_{0}=2/D_{1}$. The Ogden material model parameters are obtained by fitting to the uniaxial tensile tests as shown in Appendix A Figure A1. The obtained values for the material parameters are presented in Table 1.

### 2.4. Experimental Methods

Experiments were conducted to validate the FE model of the sensor and then to calibrate and evaluate the mechanical and sensing performance of the proposed sensor under normal indentation, using 6 and 22 mm indenters. The experimental apparatus used in this study was similar to the one used in our previous work [23]. A linear stage (T-LSR75B, Zaber, Vancouver, BC, Canada) was set to drive the indenters in the vertical direction normal to the top exterior surface of the elastically inflatable dome. A load cell (LCM201-200N, Omega, Norwalk, CT, USA) was attached in line with the indenter to measure the applied forces. The sensor setup consisted of the soft sensor as described in Section 2, combined with an air regulation system with an electro-pneumatic regulator, solenoid valve, and a pressure transducer (030PAAA5, Honeywell TruStability, Charlotte, NC, USA). This allowed a controlled air pressure within the elastically inflatable dome to alter the structural compliance of the soft sensor, as illustrated in Figure 1a. A real-time controller (myRIO 1900, National Instruments, Austin, TX, USA) was used to implement real-time closed-loop control of the pressure and data acquisition of all experimental parameters, i.e., the air pressure from pressure transducer, contact force from the load cell, and magnetic field magnitudes from the Hall-effect sensor. A PC was utilized to control the linear stage and connected to the real-time controller as a host to synchronize the clock and log the real-time data of displacement of the indenter, contact force, magnetic field, and air pressure.

The soft sensor was calibrated to correlate the change in the magnetic field output signal with the applied force across a range of pressures within the elastomeric dome. Each elastomeric dome (three different durometers) was calibrated for five different air pressure conditions, from 0 to 4 psi in 1 psi increments (five repeats at each condition). For each pressure condition, the sensor was cyclically indented vertically between 0 and 7 mm in 0.1 mm increments at a constant speed of 2 mm/s. The measurement of contact force and air pressure are both taken at the end of 0.1 mm increments. The applied external force was recorded by the load cell while displacement was recorded by the linear stage connected to the PC. The data sampling rate is 200 Hz. The air pressure was recorded by the pressure sensor and the magnetic field is recorded by the Hall-effect sensor.

**Figure 4:**
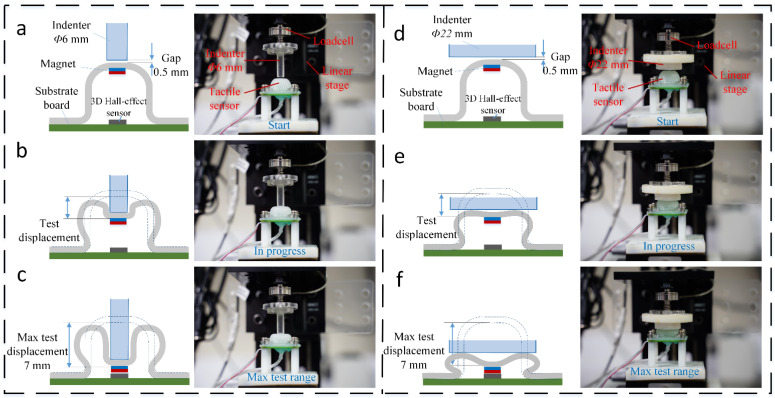
The experiment setup for the calibration of the proposed soft sensor: (**a**) the 6 mm diameter indenter lowered to a distance of 0.5 mm gap to the top surface of the elastomeric dome, (**b**) sensor then deformed by the compression of the indenter onto the dome, (**c**) indenter has reached the maximum range of the sensor, stopped, and immediately reversed upward to its initial start position. (**d**–**f**) show the same operation with a 22 mm diameter indenter.

As shown in Figure 4, before each test, the indenter was raised to leave a 0.5 mm gap between the indenting surface and the top surface of the elastically inflatable dome. This ensured a zero-valued contacting force in the beginning of this stage and avoided adhesion between the indenter and dome. During the test, the indenter was lowered slowly until contact was made with the silicone dome and then moved downward at a constant speed. An indenter of 6 mm diameter was implemented to calibrate the situation when the contacting area is small, while an indenter of 22 mm diameter was applied to calibrate the situation when contacting area is larger than the fluidic dome.

## 3. Results

### 3.1. FEA Model Validation

**Figure 5:**
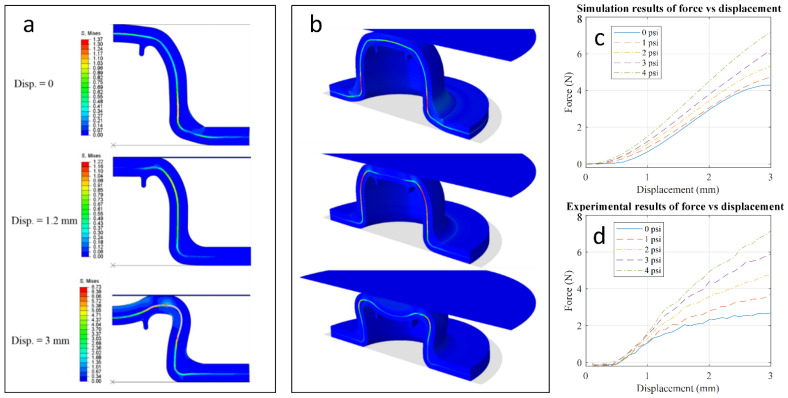
Deformation visualization of the fluidic dome with the Ecoflex 0050 soft layer pressurized at 3 psi and then indented up to 3 mm displacement using the 22 mm indenter: (**a**) two-dimensional (2D) view (legend shows the von Mises stress) and (**b**) 3D half model view. (**c**,**d**) show simulation and experimental force vs. displacement results for the fluidic dome with Ecoflex 0050 soft layer, respectively.

Figure 5a,b show the two-dimensional (2D) and 3D half-model views of the deformation of the fluidic dome with Ecoflex 0050 soft layer pressurized at 3 psi and then indented up to 3 mm displacement using the 22 mm indenter, respectively. It can be seen in Figure 5a,b that at the end of the pressurizing step (before starting the indentation, i.e., when displacement is zero), the strain-limiting layer will be in tension. As the indenter begins displacing the dome, the magnitude of the tensile stress throughout the constraining layer changes. With further increase in the displacement from 1.2 to 3 mm, deformation of the strain-limiting layer plays the dominant role, and the upper and lower silicone layers follow the deformation of this layer. With increase in the deformation of the strain-limiting layer, a dimple gradually forms under the indenter, a feature that cannot be recorded in the experiments. This dimple formation for the dome under indentation is due to the snap-through instability (buckling) deformation mechanism, which has been predicted and observed in the analytical, numerical, and experimental studies on spherical shells subjected to external concentrated, distributed, or ring loads [33,34,35,36].

Figure 5c,d show the representative simulation and experimental results for the soft sensor with the Ecoflex 0050 soft layer in the working range of up to 3 mm displacement, respectively. Taking into account the non-uniform thickness of the constituent layers due to the errors and uncertainties in the fabrication process, simulation results match the experimental ones. At 3 mm displacement and 4 psi pressure, simulation showed a 7.19 N contacting force compared to the experimental result of 7.14 N.

### 3.2. Sensor Characterization

As defined in Equation (2), external force $F_{z}$ applied on top of the inflatable dome is proportional to the displacement $d_{z}$ of the fluidic dome. The stiffness coefficient *K* is specified by recording the relations between $F_{z}$ and $d_{z}$ at different air pressures.

**Figure 6:**
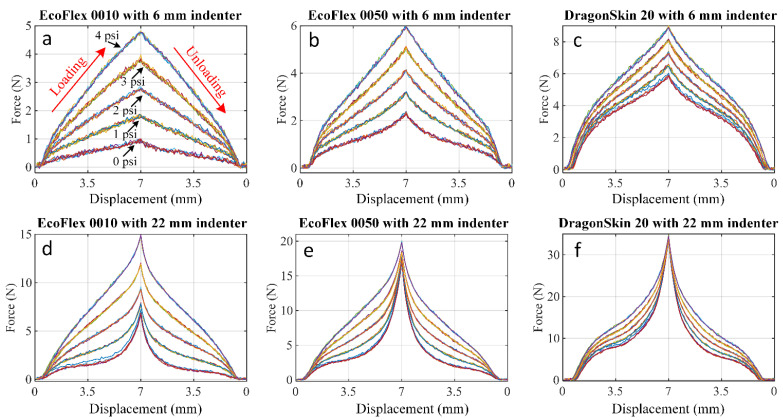
Test force-displacement results of different durometer dome samples: (**a**) Ecoflex 0010, (**b**) Ecoflex 0050, and (**c**) DragonSkin 20 when 6 mm diameter indenter was applied. Test results of different durometer dome samples: (**d**) Ecoflex 0010, (**e**) Ecoflex 0050, and (**f**) DragonSkin 20 when 22 mm diameter indenter was applied.

Figure 6 shows typical characterization results of the external force $F_{z}$ and the displacement $d_{z}$ with 0 to 4 psi fluidic pressure inflated. Both 6 and 22 mm indenters are used in the tests. The five groups of lines stand for five air pressures of 0 to 4 psi from bottom to top. Each group consists of five individual lines of test results for the same fluidic dome. The results show high repeatability of the sensor over the full pressure and indentation range investigated in this study. Over the full range of fluidic pressures and structural materials investigated, the force response at the maximum displacement of 7 mm, with 6 and 22 mm indenters, varied from 1.01 to 8.99 N and 7.19 to 34.77 N, respectively. Table 2 summarizes the maximum forces developed in the inflatable dome subjected to different levels of pressure and displaced with indenters of two sizes.

**Figure 7:**
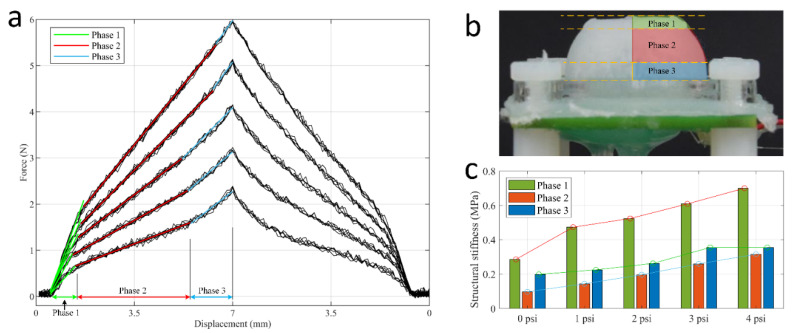
(**a**) The force-displacement data was divided into three different stages based on their gradient. The straight lines stand for linear-fit results of the trend of stiffness change. (**b**) Approximate phase division on a prototype of the inflatable dome. (**c**) Bar chart of structural stiffness factor, *K*, for Ecoflex 0050 sample by 6 mm indenter. The bars stand for linear-fit stiffness of each phase of the sensor. The stiffness of second phase of sensor increased linearly with increase in pressure.

As the fluidic pressure inside the chamber is increased, there is a small effect of inflation in the elastomeric dome, which in turn reduces the gap to the indenter. This is shown in the schematics of Figure 4e,f, and captured in the simulation results shown in Figure 5a,b. There are three phases in the force-displacement graph where the gradient of the curve (which corresponds to the structural stiffness of the sensor) changes throughout the indentation range, as shown in Figure 7a. From the ratio between force and displacement, the structural stiffness factor *K* in the *Z*-axis is calculated by:

(4) $$K_{z}=F_{z}/d_{z}$$

The structural modulus is defined by the contact section area, *A*, force, *F*, and $d_{z}/L$ of the sensor. This can be calculated using:

(5) $$E_{z}=\frac{F_{z}/A}{d_{z}/L}$$

During the process of indentation, the sensor’s structural stiffness changes due to the deformation of the dome. The first phase of indentation begins when the indenter hits the exterior surface of the elastomeric dome. This phase continues until there is a discernible change in gradient of the force-displacement data. This signifies the start of the second phase, in which the gradient and *K_z_* reduce significantly due to the buckling of the vertical walls. In the final phase, with further indentation of the dome, the horizontal and vertical walls contact and compress each other, which results in the rise of the gradient again until the end of the indentation.

The results shown in Figure 7 were obtained using a 6 mm indenter on a sensor with an Ecoflex 0050 dome. By using the 6 mm indenter in experiments, the contact area remains the same as the surface area of the indenter. In this case, the structural modulus of the 6 mm indenter test can be calculated using a constant contact section area, *A*, by Equation (5).

Using the phases defined in Figure 7a, a linear fit was applied on the force-displacement data to estimate the structural stiffness factor, *K_z_*, as well as the structural modulus in the *Z*-axis, *E_z_*. An approximate phase division on a prototype of the fluidic dome is shown in Figure 7b. The structural stiffness varied from 0.05 to 1.01 MPa across the full range of pressures and materials, as presented in Table 3 and Figure 7c. It is observed that the structural stiffness increased with the increase in the applied pressure inside the fluidic dome. It is also apparent that the relationship between the structural stiffness and pressure in the second phase is linear.

### 3.3. Sensor Calibration

A calibration process was undertaken to find the relationship of the force Z direction, $F_{z}$, as a function of magnetic field, *Bz*, and pressure, *P*, in the fluidic dome:

(6) $$F_{z}=f\left(B_{z},P\right)$$

**Figure 8:**
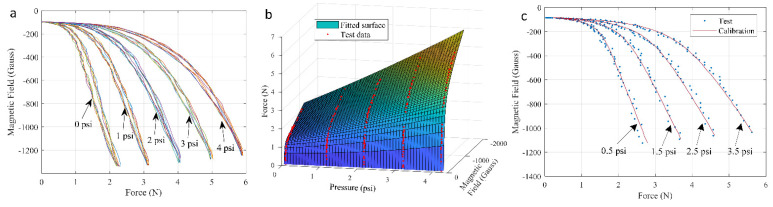
(**a**) Magnetic field (*Z*-axis, averaged) response to the indentation force for the Ecoflex 0050 sample which is pressurized at different fluidic pressure. (**b**) Fitted surface stands for the relation between force vs. magnetic field (*Z*-axis) and pressure. (**c**) Calibration by fitted surface using 1 to 4 psi (1 psi increment) on 0.5 to 3.5 psi (1 psi increment) test conditions.

This was achieved using a modified Genetic Programming (GP) approach [37,38], trained using experimental test data. The test setup (shown in Figure 1 and Figure 4) provides synchronized measurement of applied indentation load, internal air pressure, and magnetic field during controlled indentation. Figure 8a shows the results of a typical sensor sample made of Ecoflex 0050 and the relationship between force and magnetic field in the Z-direction. For the same magnitude of magnetic field, when the force range is larger, the ratio of the magnetic field to force range (the sensitivity of the sensor) is lower. When the dome was not pressurized (0 psi fluidic pressure), the force measurement range was 0~2.39 N, the magnetic field range was −99~−1337 Gauss, and a sensitivity of 517 Gauss/N was obtained. When the fluidic dome was pressurized at a maximum level of 4 psi, the force measurement range was 0~5.98 N, the magnetic field range was −96~−1247 Gauss, and a sensitivity of 192 Gauss/N was obtained.

The experimental data for indentation at 0, 1, 2, 3, and 4 psi were used as inputs to train the GP program and obtain a general formulation in the form of Equation (6). Then, a reconstruction of force, magnetic field, and fluidic pressure were conducted. Figure 8b shows the results of calibration reconstructed surface and actual pressure and magnetic field test data. The calibrated was then evaluated using distinct test data obtained during indentation at 0.5, 1.5, 2.5, and 3.5 psi, as shown in Figure 8c. It should be noted that the continuous calibration surface in Figure 8b provides data at points other than the discrete data points studied in the tests.

## 4. Discussion

This work demonstrates a conceptual soft sensor with adjustable variable compliance. Our work here has shown the feasibility of an elastically inflatable fluidic dome by pneumatic control. It also demonstrates the potential of a proposed fabrication method of constrained silicone rubber. The simulation results showed a good coherence with the experimental results and revealed the deformations of the sensor structure that could not be captured in the experiments. The experimental and simulation results show the potential of the proposed design as an adjustable soft sensor, which can be used in various soft robotics applications. While the dome was at a low air pressure condition, there was a larger discrepancy in the force measurement range between simulation and experiment. This is likely due to small imperfections or inconsistencies in the fabrication process (related to adding the dome constraint layer) that are more significant in this low pressure sensor state, but become negligible as the sensor pressure increases.

The proposed soft sensor achieved variable and controllable stiffness and compliance. Compared to the previous soft sensor [23], the elasticity of the adjustable soft tactile sensor can be much softer and much stiffer. With variable stiffness, the design of the soft sensor is now more achievable on different silicone materials, which provide more possibility on practice.

The sensor showed a variable compliance for different air pressure inside the inflatable dome as the required force for displacing the dome changed from 1.01 to 8.99 N for a 6 mm indenter. This force was also variable from 7.19 to 34.77 N for the 22 mm indenter. The force-displacement data of the sensor represented a three-phase reaction of the sensor with a linear behavior in the second phase. We also measured a variable structural modulus for the sensor in the range of 0.05 to 1.01 MPa for different domes’ material and pressure. The measurement range is potentially further expanded by applying softer or stiffer material of the inflatable dome to achieve lower measurement range but more sensitive measurement.

The limitation of the proposed sensor is that its structure is not fully soft. The substrate board and transducer are still rigid. But with the technologies of stretchable electronics, the soft sensor can be developed to the fully soft sensor. For example, a flexible and stretchable Hall-effect sensor will help to cancel the substrate board [39]. Its measuring range is adaptively changed on demand, however its highest range is constrained around 35 N when at most 4 psi air pressure is inflated. This may affect its application in some situations requiring a wide measuring range. Compared to the sensor array also using the Hall-effect transducer [40], this sensor can only detect a single point instead of a sensing map.

Potential applications of the proposed sensor are aimed at complex occasions of robots, where their contacting objects are often shifted. Adaptive and variable stiffness of this sensor helps, for example, home-caring robots to evaluate their contacting force between holding a hand and holding an iron. Sorting and logistics robots will also be more capable with the help of the variable stiffness of the proposed sensor when they are dealing with various goods. The proposed sensor could also be integrated in pneumatic robotic manipulators [41,42] systems. This variable soft sensor could improve their sensibility to different types of objects in different application occasions.

Future work on the proposed sensor will aim to optimize the sensor’s inflatable fluidic dome, which is the key component of the sensor. With improved design of the dome, it will provide a more linear and reliable measurement. This will largely increase its adaptivity and practical potential. Another direction is to apply new material and a new fabrication method to the inflatable fluidic dome to enable more air pressure, inside which will also help to increase its measuring range.

## 5. Conclusions

We have demonstrated a soft sensor platform whose structural compliance can be dynamically altered to modulate the sensor’s measurement range and sensitivity. The concept allows for safe interaction in which the sensor can change stiffness relative to applied load and the environment. The sensor consists of an elastomeric inflatable dome with an internal air cavity in which the pressure could be adjusted, resulting in a varying compliance for the dome. The relative displacement of the dome is detected by measuring the magnetic field of a permanent magnet, adhered to the dome, using a Hall-effect transducer. We found the optimized structural stiffness using different elastomeric materials and evaluated these in an experimental setup in which an indenter applied force on top of the sensor and measured the corresponding displacement for different air pressures inside the dome. FEM simulations were used to evaluate the characteristics of structural deformation under different load regimes to enable optimization of the concept. A Genetic Programming approach was then used to determine a relationship between the applied force, magnetic field, and air pressure, and this was shown to provide a robust calibration for the sensor. This novel sensor concept has relevance in a range of fields, notably the development of soft robotic manipulators. The proposed sensor potentially extends the sensibility of soft robotic manipulators in multiple applications.

This study demonstrated a new soft sensor with adjustable stiffness, compliance, and controllable measurement range. The concept of the soft sensor with a strain-limiting layer-imbedded silicone dome was validly proven. The fabrication method of the new soft sensor for tactile sensing provided a concise casting technology for rapid manufacture. Simulation and experimental results showed the performance of controllability, sensitivity, and repeatability.

## Figures and Tables

**Table 1:** The Ogden (*n* = 3) material model constants for Ecoflex 00-50 and strain-limiting layer.

Material/Coefficient	Ogden (N = 3) Material Model Coefficients								
	*μ*_1_	*α*_1_	*μ*_2_	*α*_2_	*μ*_3_	*α*_3_	*D*_1_	*D*_2_	*D*_3_
Ecoflex 0050	0.0234	3.105	0.001	3.943	−0.013	−1.219	0	0	0
Strain-limiting layer	−98.65	−0.824	21.2849	0.324	92.56	−2.227	0	0	0

**Table 2:** Maximum force (N) measurement in 6 and 22 mm indention tests for each material and pressure condition.

Material	Indenter Diameter	0 psi	1 psi	2 psi	3 psi	4 psi
Ecoflex 0010	6 mm	1.01	1.87	2.84	3.96	4.82
	22 mm	7.19	7.92	9.55	12.03	14.94
Ecoflex 0050	6 mm	2.39	3.23	4.16	5.14	5.98
	22 mm	17.53	17.65	17.74	18.63	20.06
Dragon skin 20	6 mm	6.03	6.6	7.42	8.18	8.99
	22 mm	34.08	33.86	34.08	34.26	34.77

**Table 3:** Structural modulus (MPa) of different sensor samples indented by the 6 mm indenter.

	0 psi			1 psi			2 psi			3 psi			4 psi		
Phase	1st	2nd	3rd	1st	2nd	3rd	1st	2nd	3rd	1st	2nd	3rd	1st	2nd	3rd
Ecoflex 0010	0.10	0.05	0.06	0.24	0.09	-	0.32	0.15	-	0.40	0.21	-	0.47	0.26	-
Ecoflex 0050	0.29	0.10	0.20	0.47	0.14	0.23	0.52	0.20	0.26	0.61	0.26	0.36	0.70	0.32	0.36
Dragon skin 20	0.82	0.25	0.33	0.87	0.30	0.38	0.89	0.33	0.30	1.00	0.37	0.38	1.01	0.41	0.63

‘-’ indicates that such a phase is not distinctive.

## Data Availability

Data available on request from the authors.

## Appendix A

An Instron machine (Criterion Model 43, Norwood, MN, USA) with a 1 kN load cell was used for uniaxial tensile testing at room temperature. The tests were performed at a strain rate-controlled speed of 100 mm·min^−1^. Three samples of each material type were tested. Figure A1a shows the geometry of the dog-bone sample used in the tests as per ISO standard [43] and the inset shows the black gauge points used to measure the axial strain using a digital-image-correlation (DIC) method. The engineering stress-strain curves for Ecoflex 0050 and the strain-limiting layer and Ogden model curve-fitting to those data are shown in Figure A1b.
